# Mutation Analysis in Regulator DNA-Binding Regions for Antimicrobial Efflux Pumps in 17,000 *Pseudomonas aeruginosa* Genomes

**DOI:** 10.3390/microorganisms11102486

**Published:** 2023-10-04

**Authors:** María Pérez-Vázquez, Carla López-Causapé, Andrés Corral-Lugo, Michael J. McConnell, Jesús Oteo-Iglesias, Antonio Oliver, Antonio J. Martín-Galiano

**Affiliations:** 1 Reference and Research Laboratory for Antibiotic Resistance and Health Care Infections, National Centre for Microbiology, Instituto de Salud Carlos III (ISCIII), Majadahonda, 28029 Madrid, Spain; mperezv@isciii.es (M.P.-V.); jesus.oteo@isciii.es (J.O.-I.); 2 CIBER de Enfermedades Infecciosas (CIBERINFEC), 28029 Madrid, Spain; carla.lopez@ssib.es (C.L.-C.); antonio.oliver@ssib.es (A.O.); 3 Microbiology Department-Research Institute Biomedical Islas Baleares (IdISDBa), Hospital Son Espases, 07122 Palma de Mallorca, Spain; 4 Intrahospital Infections Unit, National Centre for Microbiology, ISCIII, Majadahonda, 28029 Madrid, Spain; andres.corrallugo@gmail.com; 5 Department of Biological Sciences, University of Notre Dame, Notre Dame, IN 46556, USA; mcconnell.mike75@gmail.com; 6 Core Scientific and Technical Units, ISCIII, Majadahonda, 28029 Madrid, Spain

**Keywords:** high-risk clone, multidrug resistance, operator, repressor, resistome

## Abstract

Mutations leading to upregulation of efflux pumps can produce multiple drug resistance in the pathogen *Pseudomonas aeruginosa*. Changes in their DNA binding regions, i.e., palindromic operators, can compromise pump depression and subsequently enhance resistance against several antibacterials and biocides. Here, we have identified (pseudo)palindromic repeats close to promoters of genes encoding 13 core drug-efflux pumps of *P. aeruginosa*. This framework was applied to detect mutations in these repeats in 17,292 genomes. Eighty-nine percent of isolates carried at least one mutation. Eight binary genetic properties potentially related to expression were calculated for mutations. These included palindromicity reduction, mutation type, positioning within the repeat and DNA-bending shift. High-risk ST298, ST308 and ST357 clones commonly carried four conserved mutations while ST175 and the cystic fibrosis-linked ST649 clones showed none. Remarkably, a T-to-C transition in the fourth position of the upstream repeat for *mexEF*-*oprN* was nearly exclusive of the high-risk ST111 clone. Other mutations were associated with high-risk sublineages using sample geotemporal metadata. Moreover, 1.5% of isolates carried five or more mutations suggesting they undergo an alternative program for regulation of their effluxome. Overall, *P. aeruginosa* shows a wide range of operator mutations with a potential effect on efflux pump expression and antibiotic resistance.

## 1. Introduction

Increased expression of efflux pumps is a major antimicrobial resistance determinant in *Pseudomonas aeruginosa* [1]. This primary human pathogen is a common causative agent of life-threatening pneumonia and invasive diseases in subjects with co-morbidities and the principal pathogen associated with cystic fibrosis patients [2]. Moreover, it is able to cause serious infections in burns and wounds in immunocompetent individuals [3]. The control of infections caused by multi-drug resistant (MDR) *P. aeruginosa* isolates, i.e., resistant to antibacterial agents of at least three independent classes, is among the priorities identified by the World Health Organization [4].

Efflux pumps can enhance MDR phenotypes by reducing the cytoplasmic concentration of antibiotics to ineffective levels [5]. Most *P. aeruginosa* isolates harbor numerous antimicrobial efflux pumps [6] among which MexAB-OprM, MexCD-OprJ, MexEF-OprN and MexXY-OprM are considered those with the highest clinical importance [7]. Except for MexAB-OprM, which has been shown to have reasonably high basal activity [8], expression of these pumps is tightly repressed in order to minimize maladaptive fitness costs. Repressor proteins and their DNA binding regions have been described for many *P. aeruginosa* pumps, which follow a functionally recurrent pattern: Dimers of repressor proteins recognize cooperatively palindromic DNA repeats, i.e., their respective operators. Operator repeats overlap, or are located close to, the promoter of efflux pump genes, thus hindering the initiation of transcription by RNA polymerase. Cognate repressors and operators show an intimate molecular relationship during binding and subsequent tilting movements [9,10]. Regulatory scenarios can be global or rather pump-specific. The latter very often involves proteins encoded by genes adjacent to pump operons.

Genetic modifications that enhance the constitutive expression of efflux pumps can have clinical consequences. *P. aeruginosa* is genomically versatile and shows an outstanding capacity to acquire resistance mutations [11]. Due to the wide substrate range of these pumps, a single genetic alteration leading to pump overexpression can produce resistance to several chemically unrelated drugs. For instance, mutations in the promoter boxes, or adjacent positions, of the *muxABC-opmB* and *triABC-opmH* operons led to increased resistance in *P. aeruginosa* to all compounds linked to these pumps [12,13]. Efflux-based resistance mechanisms promote resistance to suboptimal concentrations of antipseudomonal molecules in human body and environmental niches prior to acquiring further adaptive mechanisms that synergistically increase resistance to high levels [14]. Remarkably, pumps are associated with other pathophysiological traits, such as quorum sensing, biofilm and virulence [15,16,17]. Thus, constitutive pump derepression can also alter the global clinical behavior in mutated strains.

Mutations that reduce the palindromic symmetry of operators of MDR efflux pumps can produce resistance by decreasing their affinity for the repressor by several orders of magnitude [18]. The effect of mutations in operators and repressors on binding has been described in formal structural terms [9,10]. However, the impact of such effects, ranging from full binding disruption to negligible, depends on the repressor-operator peculiarities and is hard to predict without targeted experimentation. Mutations in central repeat positions have been found to be more disruptive [19,20]. The mutation type (either transitions or transversions), the specific repeat affected and the length of the inter-repeat spacer have also been reported as relevant features causing expression switch [19,21,22]. Furthermore, mutations that alter the local DNA bending in the promoter region can influence the initiation of transcription [23,24]. Operator mutations enhancing drug efflux expression in a position-dependent manner are expected to be maintained if provided a selective advantage under clinical conditions.

Improvements in the predictive power of genome-level resistance, the resistome, can be utilized in clinical decision-making to administer the most effective therapy [25,26,27,28] and to prevent antibiotic overuse [29]. Isolates refractory to treatment commonly show constitutive expression of efflux pumps [30]. However, identification of resistance markers related with up-regulation of efflux pumps is deemed one of the most significant obstacles in the resistome field [31,32,33,34]. There is a pressing need to develop accurate prognosis strategies that discern pump operator polymorphisms with clinical value from simple mutational background.

Overall, efflux pump derepression by mutated operators is largely known and a large body of scientific knowledge on MDR pump regulation is available for *P. aeruginosa*. However, no large-scale study has screened the variety and potential clinical influence of pump operator mutations in this pathogen. Here, we have accomplished this by constructing an analysis scheme of operator mutations using information mined from the literature and the application of computational methods on a huge genome dataset. An updated and comprehensive catalog of operator mutations has been produced to analyze clinical isolates and to guide future experiments.

## 2. Materials and Methods

### 2.1. Genetic and Genomic Data Acquisition

Genes and intergenic regions for efflux pumps corresponding to the PAO1 strain genomic sequence [35] were downloaded from the *Pseudomonas* Genome Database (https://pseudomonas.com/) (Last Accession: 30 September 2023) [36]. Genbank genomes for 17,292 *P. aeruginosa* isolate samples were downloaded from the NCBI (National Center for Biotechnology Information)-Assembly database (https://www.ncbi.nlm.nih.gov/assembly) (Last Accession: 30 September 2022) by searching the term “Pseudomonas aeruginosa” [37]. Selection filters available in the server were subsequently applied and those genomes labeled as anomalous, partial, non-annotated and those that did not fully pass the taxonomy check were excluded.

### 2.2. Computational Analyses of Intergenic Regions

The architecture of operons was predicted with DOOR [38]. Transcriptional terminators in operon upstream intergenic regions were identified with TransTermHP [39]. Detection of promoters in operon upstream intergenic regions of the PAO1 reference strain was carried out with SHAPPIRE [40] using a *p* < 0.05 threshold. Promoters were also identified with Pattern Locator [41] and a {TTGACA}[2](N)[15–20]{TATAAT}[2] consensus motif. Perfect and pseudopalindromic repeats were detected in operon upstream intergenic regions in the PAO1 reference strain with the *einverted* program of the EMBOSS suite [42]. For that, a minimum of 5 repeat nucleotide length, an inter-repeat gap ≤ 10 nucleotides and ≤2 mismatches were applied. Perfect and pseudopalindromes that overlap or were located at less than 20 nt from any of the promoter boxes were selected for further analyses.

The first gene of the selected operons coding for MDR efflux pumps was identified in NCBI sample genomes by BLASTn 2.2.28+ [43] applying an ≥80% identity and ≥90% length coverage alignment. Next, the corresponding intergenic regions were extracted and identical redundant sequences removed with CD-HIT 4.6 [44] applying 100% identity and 100% alignment coverage filters. Non-redundant intergenic sequences were aligned with the wild-type sequence with Clustal Omega 1.2.1 [45] and changes in the (pseudo)palidromic regions identified by an in-house script written in Python language (available at 10.5281/zenodo. 8099826).

### 2.3. Calculation of Mutation Features

Features of mutations in (pseudo)palindrome sequences were extracted by an in-house Python script. The mutation type (transition or transversion), palindromic repeat positions and adjacency to non-palindromic positions, the number of hydrogen bonds in the DNA double helix, the repeat index in the palindrome were simplistically associated with tables. These tables were pre-filled manually and involved the nucleotide type, position within the repeat, and the relative position of repeats and promoter boxes with respect to the start codon. Extreme positions were considered the single, two and three positions at each extreme for repeats of 5–7, 8–11 and 12–13 nucleotides, respectively. The remaining intermediate positions were categorized as central. DNA curvature shifts between the wild-type and mutated sequences were calculated with bend.it 1.0 [46], applying a curvature window size of 31 nt. The definite DNA bending shift considered corresponded to the nucleotide position showing the maximal difference between PAO1 and the mutated sequence regardless if it matched the substituted position or not.

### 2.4. Repressor and Resistance Target Analyses

To identify mutations in repressors, all genomes were translated into proteomes with Prodigal v2.6.3 [47], repressor sequences identified with MMSeq2 [48] and then aligned with PAO1 protein variants with Clustal Omega 1.2.4 [45]. Resistance to fluoroquinolones (FQ) due to mutations in targets, gyrase and topoisomerase IV was detected, applying the “variant mode” of CARD 2023 [49]. Mutations associated with resistance in MexR, NalC, NalD and MexZ repressors were also acquired from CARD 2023.

### 2.5. Typing and Phylogenomic Studies

Sequence types (STs) were assigned by identification of perfect matches, i.e., 100% identity over 100% aligned length, by BLASTn. Seven genes were considered: *acsA*, *aroE*, *guaA*, *mutL*, *nuoD*, *ppsA* and *trpE*. Exact allele sequences and their combinations to define the STs were downloaded from the official MultiLocus Sequence Typing (MLST) site (https://pubmlst.org) (Last Accession: 30 September 2023) [50].

For phylogenomic analysis, 53 assembly genomes were used as input for Roary version 3.13.0 [51]. An alignment of 3780 core genes (present in >99% of isolates), comprising 3,670,255 bp, was generated. Variable positions were extracted (3,229,496 single-nucleotide polymorphisms, SNPs), and a maximum-likelihood phylogenetic tree of SNPs was constructed using RAxML version 7.0.4 [52] with a general time-reversible model and gamma correction for site variation. The phylogenetic tree and associated metadata were visualized using Figtree and iTOL.3 [53]. Core genome MLST (cgMLST) was performed, consisting of 3874 *P. aeruginosa* targets [54] provided by SeqSphere+ version 3.5.0 (Ridom, Münsten, Germany).

## 3. Results

### 3.1. Identification of Expression Elements in Core MDR/Biocide Efflux Pumps of P. aeruginosa

**Figure 1:**
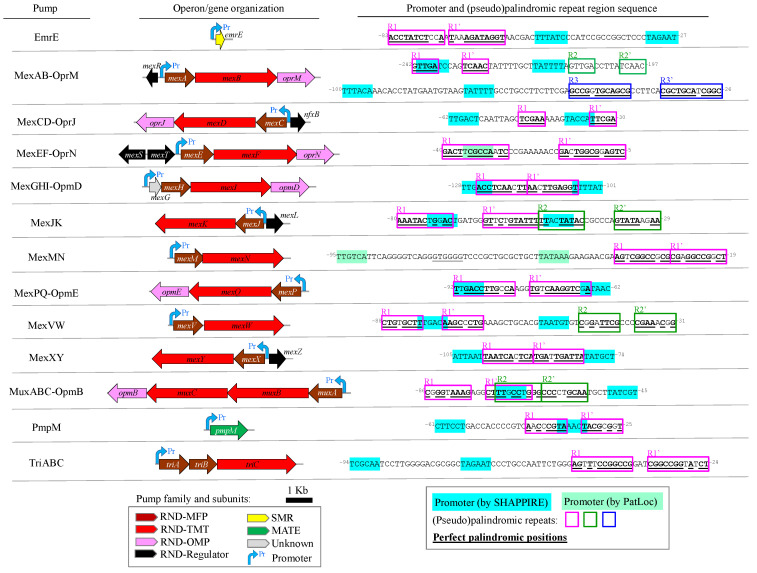
Genetic architecture and transcriptional elements intergenic sequences for gene/operons coding for antibacterial and/or biocide efflux pumps in *P. aeruginosa*. Organization of the gene/operon coding for the efflux pump is depicted in the middle. Gene lengths are at proportional scale. Efflux family, including the RND complex subunits: membrane fusion protein (MFP), transmembrane transporter (TMT), and outer membrane protein (OMP), are indicated. The location and orientation of the transcription of the pump mRNA is indicated by the promoter (Pr). The upstream intergenic 5’-3’ sequences containing promoter and potential operators, beside the corresponding spacer, are depicted rightmost. Promoters predicted by either SAPPHIRE or PatLoc software are highlighted in cyan and green, respectively. (Pseudo)palindromic sequences are boxed in fuchsia, green or blue. Perfect palindromic positions in repeats are in bold and underlined. Relative positioning to start codon are indicated as superscript numbers in the sequence limits.

To analyze potential resistance phenotypes caused by operator mutations in efflux pumps of *P. aeruginosa*, a standardized scheme was established. Researchers on this species benefit from a well-defined reference strain (PAO1) and a centralized genomic resource (pseudomonas.com) (Last Accession: 30 September 2023), which further support this microorganism as an appropriate model to study variability of efflux pump operators. Thirteen pumps were considered by their explicit association with antimicrobial and/or disinfectant efflux after exhaustive literature screening. Among them, one pump belonged to the Multidrug and Toxic Compound Extrusion (MATE), eleven to the Resistance-Nodulation-Division (RND) and one to the Small Multidrug Resistance (SMR) families (Figure 1).

Next, potential operators consisting of perfect or pseudo-palindromic repeats close to promoter sequences for the expression of these pumps were mapped onto the genome of the PAO1 reference strain. For that, sequences corresponding to the intergenic regions were inspected considering the genetic structure of their transcriptional units, either single genes or operons (Figure 1). Promoters were either experimentally reported [55] or predicted for all cases. A total of 18, ranging from one to three, (pseudo)palindromic repeats overlapping or in the vicinity of promoter boxes, at 20 nt or less, were identified for all pump transcriptional units (Figure 1). These motifs where 9.3 ± 2.3 nt (mean ± standard deviation) in length with 1.6 ± 0.7 non-palindromic positions, inter-repeat spacers of 3.5 ± 2.8 nt and distances of 2.8 ± 5.6 nt to the closest promoter box. None of these repeats were predicted as part of transcriptional terminators of flanking genes. Only palindromes for MexAB-OprM were previously reported as operators, e.g., GTTGA in *mexA* [55], and included in gene regulation resources, such as PRODORIC [56] or RegTransBase [57]. Altogether, a diverse set of (pseudo)palindromic repeats that likely act as potential operators in *P. aeruginosa* MDR pump genes were detected.

### 3.2. Most P. aeruginosa Isolates Carry Mutations in Repeat Sequences Compared with the PAO1 Strain

**Figure 2:**
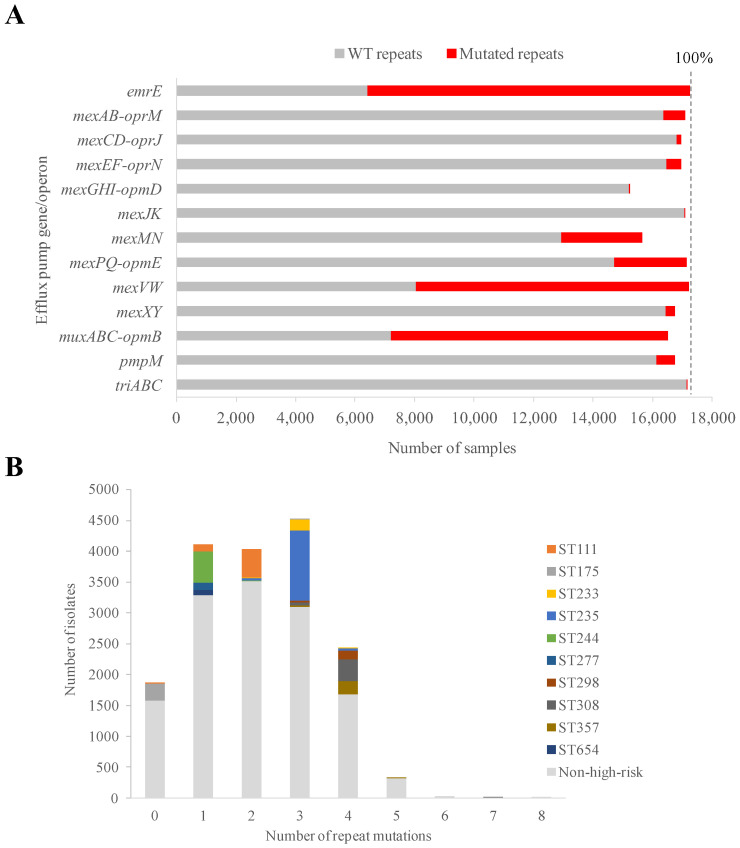
Prevalence of pumps and mutated operators. (**A**) Total number of isolates carrying efflux pump genes and repeat mutations. Vertical dashed line indicates the total (100%) number of samples (17,292). (**B**) Distribution of sample amounts according to the number of operator mutations in their genomes. High-risk clonal and non-high-risk samples are indicated.

The global occurrence of these pumps in the *P. aeruginosa* species was calculated. For that, the presence of genes coding for these pumps was searched in the genomes of 17,292 *P. aeruginosa* samples available in the Assembly public database. These mostly corresponded to independent isolates but also to some time-course experiments likely involving intra-patient isolate microevolution. Genes for all of these pumps were found in ≥88% samples (Figure 2A). Therefore, the 13 pumps may be deemed part of the extended core genome of *P. aeruginosa* or, at the very least, in the high range of the shell genome [58].

The sequence integrity of the (pseudo)palindromic repeats was checked in all pump-positive cases. Up to 144 unique SNPs were found in these potential operators (2–26 per pump, average = 11.7 ± 6.3) involving 123 unique positions (Supplementary Table S1). Although repeat symmetry may be expected to be conserved to prevent expression leakage, up to 89.2% of *P. aeruginosa* samples showed SNPs in at least one repeat sequence compared to the reference PAO1 strain. On average, 2.2 repeat mutations (6.4 mutations per Kb) were found per sample. This mutation occurrence was comparable to the ones in open reading frames coding for repressors, such as *mexR* and *nfxB* (5.9 mutations/Kb) but higher than for *nalD* (3.0 mutations/Kb) and lower than for *mexZ* (8.9 mutations/Kb) and *nalC* (11.0 mutations/Kb). The PAO1 strain was selected as the absolute negative sequence control because (i) it is the reference for countless published studies using *P. aeruginosa*; (ii) it belongs to the non-high-risk clone ST549; (iii) it showed the maximal number of palindromic positions in the repeats in our genome datasets; and (iv) it shows limited resistance due to basal expression of efflux pumps, which facilitates extrapolation between the presence of mutations and resistance phenotype in future studies. In contrast, another common strain, PA14, was rejected as a control for sequence comparison concerns as it shows resistance to a range of compounds and reduced repeat symmetry compared to PAO1. With respect to nucleotide substitution, the presence of indels or insertion sequences was almost negligible. Concerning occurrence of mutations in repeats, there was a high difference between pumps (Figure 2A). This ranged from almost full conservation of the wild-type (WT) sequence, e.g., only 21 repeat mutated samples for *mexGHI*-*opmD* (0.12% of the dataset), to general alteration, e.g., 10,843 repeat mutated samples for *emrE* (62.85% of the dataset). Mutations were further biased towards a few positions. In contrast, 63% of all repeat positions did not show any mutation, and 76% repeat positions were not mutated in more than one single isolate.

Regarding clonality, the ten high-risk clones causing the bulk of worldwide infections [59], showed their own consistent pattern shared by 81–99% of ST isolates (Figure 2B). The same mutational profiles in repeats were shared by nearly all members of the clone and the global number varied with the clone (see below). These were zero for ST175; one for ST244, ST277 and ST654; two for ST111; three for ST233 and ST235; and four for ST298, ST308 and ST357 (Figure 2B). In contrast, non-high-risk clone isolates showed a Gaussian distribution with a median of two operator mutations (Figure 2B). For statistical significance of the number of mutations in high-risk clones, the intra-ST average number of mutations for the 110 non-high-risk STs with 20 or more sequenced genomes were considered. Compared with the resulting normal distribution (*p* = 0.09, Jarque-Bera test), only ST298, ST308 and ST357 high-risk STs were in the upper quartile and were significant at the level of 0.1 > *p* > 0.05 (Z-scores: 1.34–1.43). Altogether, these data indicate that the potential effect of repeat mutations, in physiology and drug resistance, in high-risk lineages is clone-dependent.

Another question is whether the raw number of repeat mutations is globally different in environmental samples compared to those obtained in clinical-associated sites. For that, we took advantage of a sample subset in NCBI-Biosample for which the “source” field was filled and the genome available in NCBI-Assembly. We manually screened the specific source terms and were able to classify 335 environmental, 998 bloodstream, 1358 hospital and 2169 respiratory samples (see Supplementary Table S2). Environmental samples harbored a number of mutations (2.41 ± 1.32, average ± standard deviation), similar to hospital isolates (2.38 ± 1.28) but significantly higher than bloodstream (2.17 ± 1.35, *p* = 0.006, two-tailed Student’s *t*-test) and respiratory (2.12 ± 1.25, *p* < 0.001) isolates.

As the former calculation is based on the naïve assumption that all mutations are equally important, the enrichment in clinical sources was interrogated separately. The mutation mexP_R1’_C10T (see below for mutation nomenclature) was 2.3-fold enriched in respiratory samples (*p* < 0.001, Fisher’s exact test) and mexE_R1_T4C 2.8-fold enriched in bloodstream samples (*p* = 0.05). Moreover, mexP_R1’_G2A and mexX_R1_C7T were found in a large number of respiratory samples, 27 and 14, respectively, and pmpM_R1’_G4A in 10 hospital samples, while none in environmental ones. Overall, these data suggest that, if all repeat mutations exert an effect, repeat mutations may be required to survive under the changing environmental conditions. Other repeat mutations are, however, linked to healthcare settings.

### 3.3. Repeat Mutations Show Diverse Genetic Properties

To facilitate analyses, a nomenclature to term the mutations in these repeats was created. For simplicity, the mutation definition consisted of: (1) just the first gene of the pump operon; (2) the letter “R” for the repeat plus the repeat pair index and the addition of apostrophe (‘) for the downstream repeat of the repeat pair; and (3) the base substitution including the original nucleotide besides the position index within the repeat and the new nucleotide. These parameters were split using the underscore symbol. For instance, muxA_R1′_C1T indicated a C-to-T substitution in the first position of the downstream repeat of the repeat pair concerning the operon coding for the MuxABC-OpmB pump.

**Figure 3:**
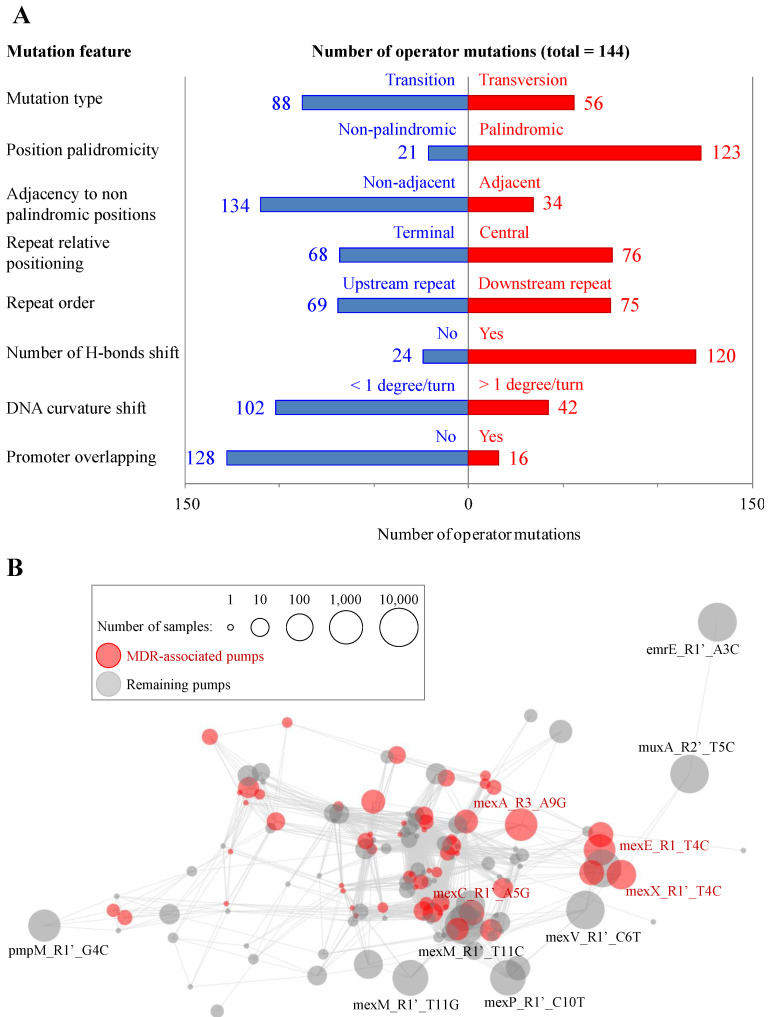
Operator mutation property correlations and mutation groups. (**A**) Occurrence for binary properties of operator mutations. (**B**) Organization of the mutational space in upstream repeats of *P. aeruginosa* efflux pumps. Nodes correspond to independent mutations. Edges (in light gray) correspond to links between mutations sharing exact values for seven, i.e., all but one for those considered, between mutation pairs. Nodes from the four principal pumps (see Introduction) are in red while the remaining ones are in gray, and labeled when involved ≥50 and ≥500 samples, respectively. The sphere size is proportional to the number of samples carrying the mutation.

To investigate the possible existence of recurrent mutational patterns, eight binary properties reflecting genetic traits were calculated for all repeat SNPs. These were based on literature antecedents concerning transcriptional regulation and on basic DNA knowledge. These properties were: (i) mutation type, either transition or transversion; (ii) palindromicity of the mutated position in the wild-type sequence, either yes or no; (iii) adjacency to WT non-palindromic position, which would create a two non-palindromic base tract; (iv) relative positioning within the repeat, either central or terminal (see Materials and Methods); (v) the exact repeat mutated in the pair, either the upstream or the downstream (labeled with an apostrophe) one, since they do not act equally during the cooperative binding process [22]; (vi) changes in the number of hydrogen bonds (i.e., three for G-C and two for A-T) between complementary bases in the DNA double strand; (vii) the maximal DNA bending shift caused by the mutation (either over one degree per turn or below); and (viii) the overlapping respect to the −35 or the −10 promoter boxes (either “yes” or “not”). These properties showed different degrees of unevenness (Figure 3A). For instance, positioning of the mutation within the repeat was relatively balanced (53% central vs. 47% terminal) whereas promoter overlapping of the mutated position was limited with respect to non-promoter positions (11 vs. 89%, respectively). The whole list of repeat mutations, property values and linked genetic and epidemiological (ST, geotemporal data) information is available as Supplementary Table S1.

According to their profiles, the 144 mutations detected in the sample dataset grouped into 58 single patterns. The network structure of the mutation landscape, linked by bridges sharing exact binary values or for all but one feature, consisted of a continuous of network communities instead of well-separated large clusters (Figure 3B).

Some particular feature combinations are indicative of mutation relevance. On the one hand, 29 mutations involved central repeat positions, reduced the palindromicity of the original repeat pair, changed the number of hydrogen bonds between DNA strands and produced DNA bending shifts over one degree per turn. There is therefore cumulative evidence that these mutations reduce regulator affinity. On the other hand, 45 mutations were in terminal and/or non-palindromic positions besides showing no promoter overlapping nor DNA bending shifts, suggesting weak or no relevance in regulation. The remaining mutations were found in intermediate situations with respect to these two extreme archetypes.

Another important factor for mutations is their occurrence in the dataset of sequenced samples. Five mutations were highly distributed (36–63% samples, and in many unrelated STs) in *P. aeruginosa*: emrE_R1_A1G, mexM_R1′_T11C, muxA_R2′_T5C and mexV_R1′_C6T. These were responsible for high mutant isolate prevalence stated in Figure 2A. Besides, eight mutations were in 180–2686 (1–15%) samples, and the remaining 131 mutations were in 100 or less genomes (see Table 1 for top-20 prevalent operator mutations).

The co-occurrence of repeat mutations and repressor amino acid changes was also evaluated. In particular, 14 repressor point mutations explicitly linked to drug resistance in the CARD 2023 database were considered. While these key residue mutations co-existed with up to 26 repeat mutations (Supplementary Table S3), only in three cases (the MexR-A156T change with mexA_R1_T2C, mexA_R3_G5A or mexA_R3_A9G) involving the total of five isolates, were identified. This suggests repeat and repressor mutations leading to resistance may represent rather redundant mechanisms. Thus, they would not tend to be co-selected in healthcare settings.

### 3.4. Repeat Mutations Involve Relevant Pumps in High-Risk Clones

In 123 (85.4%) of 144 unique SNPs, the mutation reduced the number of palindromic bases present at a pair of the repeats. This may reduce affinity by repressor and consequently enhanced resistance due to pump upregulation, as stated above.

**Figure 4:**
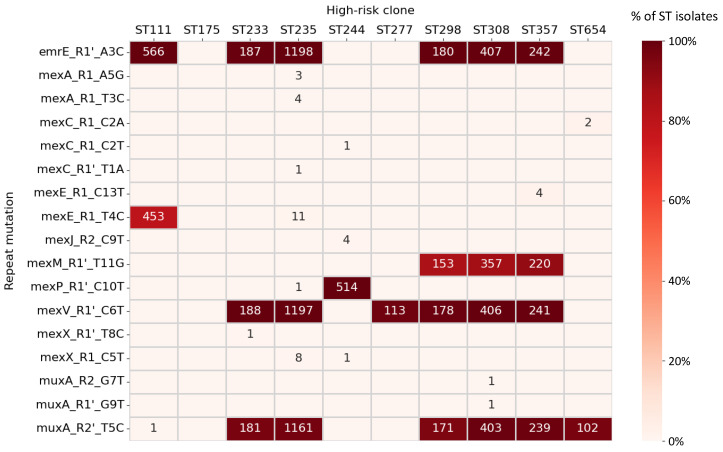
Prevalence of repeat mutations involving efflux pumps in high-risk clones. Heatmap shows in a color-ranked manner the percentage of ST isolates carrying the repeat mutation. Raw numbers of samples carrying the mutation are indicated inside cells. Only non-zero values are shown. Only repeat mutations globally found in two or more isolates, including at least one in high-risk clones, were considered.

SNPs with potential regulatory impact may be of particular clinical relevance in the case of high-risk clones. Sixteen repeat mutations were detected in at least one isolate from high-risk STs (Figure 4). Of importance, all but one in these mutations reduced repeat palindromicity whereas the remaining one imposed DNA bending above one degree per turn. Expectedly, three of the mutations very common in high-risk clones were also globally spread mutations in *P. aeruginosa*: mexM_R1′_T11G, mexV_R1′_C6T and muxA_R2′_T5C.

SNPs involving the four principal efflux pumps associated with MDR (see Introduction) were prioritized for analysis in detail. The most remarkable hallmark case was the mexE_R1_T4C mutation. This change exhibited important genetic features and was present in 79.9% ST111 isolates while nearly absent in any other ST. Searching for signs of selection, we focused the examination of this mutation in ST111 samples from a single country (USA), the one with more ST111 samples with available geotemporal metadata. Samples with the mutation isolated in the 1979–2017 period were only 15.6% of the total (5 out of 32 cases) but increased to 84.7% (83 out of 98 cases, *p* < 0.001, Fisher’s exact test) thereafter. This rejects the null hypothesis of a constant mutation prevalence and suggests active replacement of ST111 isolates by the mutated subpopulation, concomitantly with the increment of the number of ST111 sequenced genomes in this country. In contrast, mexM_R1’_T11G, abundant in ST298, ST308 and ST357 high-risk clones, did not experience temporal changes, and thus the null hypothesis remained valid. Surprisingly, eleven ST235 cases (0.9% in the clone) also carried the mexE_R1_T4C marker that, after sample metadata inspection, mostly corresponded to clinical samples isolated in Argentina between 2010 and 2019 [60].

Other mutations involving MDR pumps also showed a local distribution. This was the case for mexA_R1_T3C detected in four clinical ST235 isolates from Boston (2015–2016), mexC_R1_C2A in two ST654 Slovenian isolates found in wastewater in 2014, and mexX _R1_C5T in nine ST235 isolates, eight from Australian healthcare wastewaters in 2017 and one causing a low-respiratory tract infection in Portugal. In this light, Australian samples from the latter case showed enhanced ciprofloxacin and benzalkonium chloride resistance, two substrates of the MexXY-OprM pump, removable by the presence of pump inhibitors [61]. Many of these mutations showed relevant feature combinations according to our scheme that suggest expression dysregulation of the cognate pump.

Clones associated with cystic fibrosis [62] were also evaluated. No single repeat mutation was found in the 24 ST649 (Australian Epidemic Strain 1, AES-1) isolates of our dataset. In contrast, all (n = 165) ST146 isolates of the Liverpool epidemic strain (LES) showed the mexP_R1′_C10T mutation. Among them, two strains from UK lung samples (named 89570 and PHELES14, collected in 2008 and 2012, respectively) also carried the presumably relevant mexA_R1_G4T change. This mutation was only found in five more isolates from other unrelated STs, countries and diseases since 2019 (Supplementary Table S2). In addition, the mexC_R1′_C3T change was found in cystic fibrosis studies concerning ST801 during Australian sampling between 2002 and 2012 [63]. Finally, mexX_R1_A6G was detected in a patient longitudinal study involving a ST2192 isolate infection [64].

Other central mutations were linked to non-high-risk STs in a (near) exclusive way, suggestive of potential clinical emergence. For instance, the mexX_R1_C7T polymorphism was mostly associated with ST508 isolates, which caused a recent outbreak in China [65]. Other changes involved repeat central positions and associated with STs rarely found in clinical surveys. Examples for these were mexG_R1_C5A in ST1248, mexM_R1′_A3G in ST701 and pmpM_R1′_G4A in ST3218 (Supplementary Table S4).

The presence of repeat mutations can be further screened using resistance databases although the size of these resources is small compared to genomic ones. This was carried out focused on resistance to FQ, a common substrate for efflux pumps. Mining the NCBI-Biosample resource rendered 182 genome sequenced *P. aeruginosa* isolates for which antibiograms including FQ were available. Resistance phenotypes were compared with resistome predictions involving the commonest FQ resistance determinants, i.e., point mutations in primary targets gyrase and topoisomerase IV, using an updated version of an important protocol, CARD 2023. Noticeably, for the four ST111 isolates in this dataset, the two highly-resistant isolates (minimal inhibitory concentrations [CMIs] of 8 mg/L for ciprofloxacin and 8 mg/L for levofloxacin) carried both target mutations and mexE_R1_T4C. However, the intermediate-resistant isolate (CMI: 2 mg/L and 4 mg/L, respectively) carried only the repeat mutation. Finally, the susceptible isolate (CMI: 1 mg/L for both FQ) carried neither target mutations nor the repeat mutation one. As MexEF-OprN is involved in moderate FQ resistance [66], these four phenotypes reinforce the notion that mexE_R1_T4C may be important for the adaptation of the ST111 high-risk clone to healthcare environments. In addition, another two repeat mutations, mexA_R3’_C6T and mexC_R1’_T2C, concerned pumps involved in FQ extrusion [67] and were exclusively found in FQ-intermediate resistant isolates with, however, predicted FQ-sensitive resistomes. It should be mentioned that CARD 2023 has not implemented the efflux-resistance model yet [49], which warrant all these mutations should be experimentally checked.

### 3.5. Several Non-High-Risk ST Isolates Harbor Five or More Efflux Pump Repeat Mutations

The potential effects of accumulated mutations in repeats were also analyzed. Strikingly, up to 259 samples (1.5% of the total) carried five or more mutations in these repeats (Z-score > 2.24, *p* ≤ 0.01, with respect to the ST-averaged Gaussian distribution mentioned above). These included 42 STs with two or more samples sharing the same mutational pattern. These involved 23 repeat mutations that overlapped between STs to different extents. Noticeably, most of these mutations located in the downstream repeat of the repeat pair. The phylogenetic relationship of these isolates and with high-risk clones was studied at a genomic level. For that, 42 strains representative of these multi-mutated STs were selected for analysis, besides isolates of the ten high-risk clones and the PAO1 reference strain.

**Figure 5:**
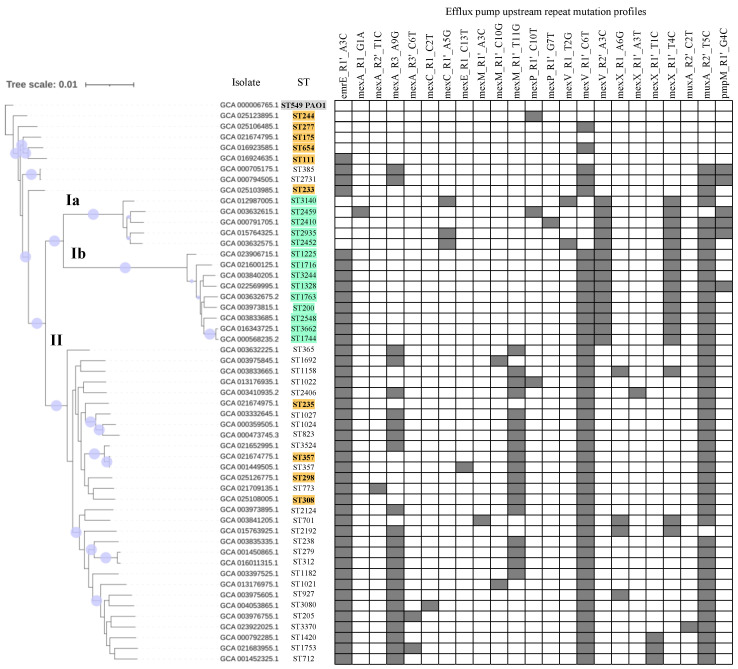
Phylogenomic analysis of isolates carrying five or more repeat mutations. A phylogenomic tree is depicted on the left. Variable positions in *P. aeruginosa* core genes of the total of fifty-three assembly genomes were aligned as indicated in Methods. Lilac solid circles on branches denote bootstrap values with greater than or equal to 99% support. Representative genomes for forty-two STs showing five or more repeat mutations, besides representative isolates for the ten high-risk clones (in orange) and reference PAO1 strain (in gray) were considered. Clade Ia and Ib isolates are in green. The Genbank entry and ST are indicated. Profiles denoting the presence (filled cells) or absence (empty cells) for mutations in repeats are shown on the right.

The phylogenomic tree revealed two salient clades (Figure 5). In addition, a series of strains with two-to-none mutations, including PAO1 and five high risk-clones, located close to the root. One of the clades, termed clade I (in orange in Figure 5), was further subdivided into two sub-branches (clades Ia: 5 isolates, and Ib: 8 isolates). As a hallmark, the whole clade I contained mexV_R2’_A3C in exclusivity and was also associated almost perfectly with the presence of mexX_R1’_T4C. Furthermore, the subclade Ia harbored mexC_R1’_A5G (three isolates) and pmpM_R1’_G4C (three isolates) but no isolates in this subclade showed the common emrE_R1’_A3C change. Both subclades of clade I typically include single, double and triple locus variants of MLST alleles. The clade II included 26 isolates plus four high-risk clone representatives (ST235, ST298, ST308 and ST357). This clade was enriched in isolates carrying the mexA_R3_A9G and mexM_R1’_T11G changes, which are fully absent in clade I. In addition, some mutations were ST-specific. For instance, mexP_R1′_G7T was only found in ST2410 (clade Ia) while mexA_R2’_T11C and mexM_R1’_A3C were detected in ST773 and ST701 (both in clade II), respectively.

All clades and subclades here were fully unrelated to each other, according to bootstrap support and the absence of MLST allelic sharing. This indicates that clade I is not commonly associated with hospital-related infections. Altogether, STs carrying an unusual amount of repeat mutations were phylogenetically associated and the subsequent lineages linked to particular mutational profiles. Findings also suggest that most repeat mutations are broadly distributed, potentially ancient and acquired in an additive manner while other are more exclusive and recent.

## 4. Discussion

*P. aeruginosa* is an intrahospital pathogen prioritized by the World Health Organization, and its MDR phenotype relies, to some extent, on numerous drug efflux pumps. However, the role of operator variability on regulation of these determinants had not yet been fully approached. Such alterations may lead to high and/or constitutive efflux pump expression with direct clinical implications. To our knowledge, this is the first article that critically approaches this topic in depth. Previously published data concerning operons coding for *P. aeruginosa* efflux pumps have been integrated to create an analyses framework. This framework consists on eighteen (pseudo)palindromes of thirteen pumps and eight mutation genetic features. This scheme has been further applied to provide a comprehensive catalog of mutations in repeats compatible with operator activity using a complete and updated genomic landscape of this microorganism. Thus, while antecedents mostly concentrate on specific isolates or pumps, this study increases the operator mutation analysis to a huge pangenomic representation of a bacterial species. By introducing a 8mer scheme for mutation analysis unavailable so far, we have observed that the 144 SNPs found in (pseudo)palindromic repeats showed extraordinary variation in genetic and epidemiologic terms. This fact likely results in a wide phenotypic relevance for this type of genomic determinant, likely ranging from causing MDR phenotype to simple genetic noise with marginal, if any, effect on pump expression. Notably, 85% of mutations reduced the original repeat symmetry and, among them, 29 mutations located in central repeat positions, involved changes in the hydrogen bonds between both DNA strands, over one degree per turn DNA bending and affected the four most important pumps classically associated to MDR. Thus, these data suggest these 29 changes are strong candidates that potentially alter the tight structural–functional relationship between repressor and operator, and to produce phenotypes of resistance against antibiotics and biocides linked to these pumps. Moreover, as efflux pumps play several roles, changes in expression may concomitantly affect other central aspects. For instance, repeat mutations could alter virulence and biofilm development in different ways considering the high efflux pump redundancy observed in this pathogen. For these reasons, mutations leading to upregulation of distinct pumps sharing activities, e.g., fluoroquinolone resistance, may be more permissive for some pumps than for others.

Original sequences for all potential operators detected showed two or fewer natural mismatches indicating selective pressure to maintain considerable palindromicity. However, most *P. aeruginosa* isolates carried at least one repeat mutation with respect to the non-virulent and drug susceptible reference PAO1 strain. This may mirror the inherent enrichment of genomic databases towards intrahospital specimens. Four mutations were independently detected in ≥36% samples of the species, which may be involved in the reported extended tolerance to biocides reported for *P. aeruginosa* [61]. These global mutations affected to the MexPQ-OpmE pump, related to survival against copper, detergents and industrial preservatives [68] and to the MuxABC-OpmB pump, related to tolerance to chlorine [69]. These also include MexVW, linked to resistance to fluoroquinolones among other antibiotics and dyes, which may utilize pore units coded by other Mex operons with higher basal expression [70]. The fact that many pumps are involved in extrusion of disinfectants widely used in the healthcare environments, these biocides may have behaved as a permanent selective factor for the observed operator mutations. Thus, the role of wide mutations in the general *P. aeruginosa* growth versatility warrants further investigation.

Data deployment allowed us to identify non-coding nucleotide changes associated with challenging isolates, unrelated with those changes reported in regulatory proteins. Genetic and epidemiological data were cross-referenced taking advantage of available genomic metadata for this microorganism. Repeat changes were investigated under different clinical scenarios. These included global high-risk clones (and their local sub-lineages), cystic fibrosis studies and other STs deemed non-high-risk. This may cast light on the fundamental knowledge of the origin of high-risk and emergent clones that show anomalous resistance behavior according to standard resistome tools. Perhaps the most noticeable finding is that the mexE_R1_T4C change is essentially a signature of ST111 samples, progressively enriched in this ST and in bloodstream samples, and with a presence compatible with antibiogram data. This mutation may have therefore ancestrally contributed to turn this clone into a persister and then into a high-risk clone. The deletion of the region containing these repeats causes a four-fold expression upregulation of the pump genes, supporting these are actually the binding site for a repressor unknown so far [71]. Some high-risk isolates, and from other lineages, showed relevant mutations in operators and operator-like sequences associated with the relevant MexAB-OprM, MexCD-OprJ and MexXY efflux pumps. These may alter the binding of explicit repressor of these pumps, i.e., MexR/NalC/NalD, NfxB and MexZ, respectively. Moreover, a large body of evidence supports MexAB-OprM and MexCD-OprJ being involved in resistance to biocides such as chlorhexidine, benzalkonium chloride and triclosan [72]. This suggests that some emerging isolates may, due in part to repeat mutations, increase survival in medical centers and cause outbreaks. In stark contrast, regulatory mechanisms may tolerate other repeat mutations leading to phenotypical irrelevance.

Concerning pump derepression, a central question is what are the differences between operator mutations and those mutations causing amino acid changes in repressor proteins, which are already integrated in conventional resistome tools. Regarding repressors, a deletion of the whole repressor gene, specific residue changes or small indels that diminish protein self-interaction or operator affinity dramatically enhance pump expression [73]. Instead, mutations in operators are obviously not interpretable in amino acid terms but in palindromicity-driven affinity by functional repressors. Moreover, general and even local pump repressors regulate a set of genes beyond the efflux pump [74,75]. In contrast, operator mutations may not produce gross effects on bacterial physiology by producing a finer and precise pump expression tuning. This may render resistance levels comparable to those caused by repressor changes but with moderate fitness penalty even in multi-pump compatible dysregulatory regimes. In fact, 1.5% of sequenced *P. aeruginosa* samples, from numerous independent STs, showed five or more mutations in regions compatible with operator activity. These are grouped into two major phylogenomic clades that include four high-risk clones. This simultaneous mutation pattern suggests a full reconfiguration of the expression regime of the drug effluxome, which must be analyzed in detail.

A limitation of our study is that it has followed a pure theoretical approach. However, we have accurately revised antecedents in literature concerning pump overexpression and resistance due to decrement of operator symmetry. All hypotheses generated here must be thoroughly validated by experimentation. However, we hope they are instrumental in guiding future conclusive experiments. These may concern the fundamental evolutionary origin of high-risk, emerging and outbreak clones that show anomalous resistance phenotypes in this prioritized pathogen. Then, operator mutations may be also considered as part of the genetic building blocks leading to high-level resistance [76]. This knowledge may reduce false negatives of new generation resistome tools and subsequent therapeutic failure leading to increment in the cost treatment, sequelae and death. Thus, we envisage future resistome databases will contain operator mutations proven as resistance determinants using standardized observations. In this regard, it should be kept in mind that efflux pump-associated markers increase resistance only modestly but to an array of unrelated compounds. Experiments, involving phenotypic changes in isogenic point mutants, to check the principal findings arising from our results are ongoing.

## Figures and Tables

**Table 1:** Top-20 repeat mutations by prevalence in genome sequenced samples.

Mutation	Number of Isolates	Change Type	Switch in the Number of H-Bonds	Position Palindromicity	Adjacency to Non-Palindromic Bases	Nucleotide Repeat Positioning	Repeat Order	Promoter Overlapping	DNA Bending Shift
emrE_R1’_A3C	10838	Transversion	Yes	No	Yes	Central	Downstream	No (upstream)	1.783
mexM_R1’_T11C	10774	Transition	Yes	Yes	No	Terminal	Downstream	No (downstream)	−0.412
muxA_R2’_T5C	9082	Transition	Yes	No	Yes	Central	Downstream	No (box spacer)	1.096
mexV_R1’_C6T	9018	Transition	Yes	Yes	Yes	Central	Downstream	No (box spacer)	0.534
mexP_R1_G9A	6264	Transition	Yes	Yes	Yes	Central	Upstream	No (box spacer)	1.684
mexM_R1’_T11G	2686	Transversion	Yes	Yes	No	Terminal	Downstream	No (downstream)	0.522
mexP_R1’_C10T	2438	Transition	Yes	Yes	Yes	Terminal	Downstream	No (box spacer)	−1.561
mexA_R3_A9G	656	Transition	Yes	Yes	No	Central	Upstream	No (downstream)	−0.306
pmpM_R1’_G4C	593	Transversion	No	Yes	Yes	Central	Downstream	No (downstream)	−0.531
mexE_R1_T4C	475	Transition	Yes	Yes	Yes	Central	Upstream	No (downstream)	1.025
mexX_R1’_T4C	213	Transition	Yes	No	No	Central	Downstream	No (box spacer)	0.846
muxA_R2’_C1T	200	Transition	Yes	Yes	No	Terminal	Downstream	No (box spacer)	−1.571
mexV_R2’_A3C	180	Transversion	Yes	Yes	No	Central	Downstream	No (downstream)	−2.192
emrE_R1’_G10A	94	Transition	Yes	Yes	No	Terminal	Downstream	No (upstream)	0.205
mexC_R1’_A5G	51	Transition	Yes	Yes	No	Terminal	Downstream	No (downstream)	0.758
mexX_R1_A6G	49	Transition	Yes	Yes	Yes	Central	Upstream	No (box spacer)	−0.794
mexX_R1_C7T	46	Transition	Yes	No	No	Central	Upstream	No (box spacer)	1.549
mexP_R1’_G2A	42	Transition	Yes	Yes	Yes	Terminal	Downstream	No (box spacer)	−0.48
mexC_R1_G3T	33	Transversion	Yes	Yes	No	Central	Upstream	No (box spacer)	−0.838
mexC_R1_T1C	32	Transition	Yes	Yes	No	Terminal	Upstream	No (box spacer)	−0.256

## Data Availability

The authors confirm all supporting data, code and protocols have been provided within the article or through supplementary data files. Supplementary files are also available as Open Data on Zenodo (10.5281/zenodo. 8099826).

## Supplementary Materials

The following supporting information can be downloaded at: https://www.mdpi.com/article/10.3390/microorganisms11102486/s1, Table S1: Mutations in repeats associated with drug-efflux pump operons of *Pseudomonas aeruginosa*; Table S2: List of samples with source metadata utilized in this study; Table S3: Co-existing repeat mutations and repressor changes linked to resistance in the CARD 2023 database; Table S4: ST and geotemporal data of isolates carrying repeat mutations.
